# Fat free mass and obesity in relation to educational level

**DOI:** 10.1186/1471-2458-9-448

**Published:** 2009-12-04

**Authors:** Elina Seppänen-Nuijten, Marjaana Lahti-Koski, Satu Männistö, Paul Knekt, Harri Rissanen, Arpo Aromaa, Markku Heliövaara

**Affiliations:** 1 National Institute for Health and Welfare, Mannerheimintie 166, 00300 Helsinki, Finland; 2 Finnish Heart Association, Oltermannintie 8, P.O.Box 50, 00621 Helsinki, Finland

## Abstract

**Background:**

The aim of the study was to describe the body composition of Finnish adults, especially by education, and to investigate whether fat-free mass (FFM) can explain educational gradients relating to body mass index (BMI) and waist-to-hip ratio (WHR).

**Methods:**

Data for this cross-sectional study were based on data collected in 2000-2001 for the Health 2000 Survey. Of the nationally representative sample of 8,028 Finnish men and women aged 30 years and older, 6,300 (78.5%) were included in the study. Body composition measurements were carried out in the health examination, where FFM was assessed with eight-polar bioelectrical impedance analysis. Questions on education were included in the health interview.

**Results:**

The mean FFM varied by education in older (≥ 65 y.) men only. In the middle-aged group (30-64 y.), highly educated men were less likely to belong to the lowest quintile of FFM (OR 0.67, 95%CI 0.48-0.93) compared with the least educated subjects. The level of education was inversely associated with the prevalence of high BMI and WHR in middle-aged men. In women, the respective associations were found both in middle-aged women and their older counterparts. Adjustment for FFM slightly strengthened the associations of education with BMI and WHR.

**Conclusions:**

The association between education and FFM is weak. Educational gradients of high BMI and high WHR cannot be explained by FFM.

## Background

Obesity is characterized by excess adipose tissue. Quantification of adipose tissue mass can be achieved by a number of laboratory methods including underwater body density measurement, dual energy X-ray absorptiometer (DEXA) and magnetic resonance imaging [1-3]. These methods require costly equipment and are difficult to implement in epidemiological studies. Some exceptions, such as bioelectrical impedance (BIA) exist[4]. To date, however, population surveys using BIA are scarce. Body weight adjusted for stature (body mass index) is yet commonly used as a surrogate for body fat content in population surveys[5,6].

A strong inverse association between overall obesity, as defined by body mass index (BMI), and socio-economic status, mostly assessed by educational level, is well defined in affluent populations [7-14]. In general, this association does not seem to be as consistent for men as for women [10,12,14,15].

To date, obesity measurements other than BMI have seldom been used to examine the relationship between socio-economic status and obesity. In a few studies waist circumference or waist-to-hip ratio (WHR), as measures of abdominal obesity, has been shown to be inversely associated with education both for men and women [14,16-21] or only for men[22].

Because BMI cannot distinguish between fat mass and fat-free mass (FFM), it is not known from which component the educational gradient of BMI is composed of. Although FFM has been shown to be positively associated with BMI [23] and also weakly associated with WHR, [24]. it remains unknown, whether the educational differences are due to differences in muscularity or in fatness.

Obviously, people carrying out more physical activity have higher FFM than less active people. To our knowledge, however, there are no studies on the association between FFM and education. It has been observed that high socio-economic status (SES) or high level of education is associated with less job-related physical activity[25]. Therefore, on one hand, we hypothesized that less educated people might be more muscular and have higher FFM than people with higher education due to manual work. However, as the typical jobs today are physically much less demanding than the working conditions some decades ago [26], these possible associations might vary by age. On the other hand, education has been shown to be associated with more frequent leisure time physical activity, [25,27] suggesting that due to being more active at leisure time subjects with high SES could have higher FFM than their counterparts in lower SES groups.

The aim of the present study was to describe body composition of Finnish adult population, especially using FFM assessed by BIA as an indicator, and its relation to educational level. Our further aim was to investigate the possible effects of the variation in FFM on the educational gradients in BMI and WHR, i.e. whether FFM can explain the educational gradients.

## Methods

### Participants

The Health 2000 Survey is a comprehensive cross-sectional health interview and health examination survey that was conducted in Finland from 2000 to 2001. It is based on a two-stage stratified cluster sampling design. The sample consisted of 8 028 subjects aged 30 or older representing the Finnish population[28,29]. The study protocol was approved by the Ethics Committee for Research in Epidemiology and Public Health.

Of the original sample, 1 674 subjects did not attend a health examination, resulting a participation rate of 79.1%. After excluding 36 pregnant women and 18 subjects for having data missing for education, the final number of subjects in this study was 6 300 (2 866 men and 3 434 women), 78.5% of the original sample. BMI was measured for 6 277, WHR was measured for 6 242 and FFM for 5 789 subjects.

The health interviews were conducted mainly at the respondents' home. If a subject's interview was not satisfactory, a supplementary interview was conducted or then a questionnaire was sent later. During the interviews the respondents were handed an information leaflet and an informed consent form that was returned after signing. A few weeks after the interviews, the subjects attended a comprehensive health examination at a local health care centre.

### Measurements of body composition

As a part of the health examination, body composition measurements were carried out by trained personnel[28,29]. All examinees had been asked to come to the examination after not eating for at least four hours and without drinking anything else than water on that day. In addition, they had been asked to avoid heavy physical activity before the examination.

FFM was assessed with an eight-polar tactile-electrode impedance-meter (InBody 3.0, Biospace, Seoul, Korea). This instrument measures the resistance of the arms, trunk and legs at frequencies of 5, 50, 250 and 500 kHz and makes use of eight tactile electrodes: two are in contact with the palm and thumb of each hand and two with the anterior and posterior aspects of the sole of each foot. Bioelectrical impedance analysis was not carried out for patients with a pacemaker.

Weight was measured to the nearest 0.1 kg and height to the nearest 0.5 cm with light clothes and without shoes. In most cases (92%), weight was measured as part of the bioelectrical impedance examination with a spring scale. In some cases weight was measured with a portable spring scale (3%) because delivery of new bioelectrical impedance instruments to some health centres was delayed. Weights and heights were self-reported (5% and 8%, respectively), if appropriate measuring was impossible for any reason. BMI was calculated as weight (kg) divided by the square of height (m^2^).

Waist circumference was measured midway between the lower rib margin and the iliac crest. Hip circumference was measured at the level of the widest circumference, which is usually the widest level of the iliac crest. Both waist and hip circumferences were rounded up to the nearest 0.5 cm. WHR was calculated as waist circumference divided by hip circumference.

The quality of body composition measurements was estimated by quality assurance and quality control measures according to the study protocol[28]. The agreement (intra-class correlation coefficient) between operators for WHR was 0.88. For waist circumference, it varied from 0.94 to 0.99 and for hip circumference it varied from 0.94 to 0.98. The intra-class correlation coefficient for 6-month repeatability was 0.90 for waist circumference and 0.89 for hip circumference. The validity of segmental multi-frequency bioelectrical impedance was estimated by comparing segmental distribution of FFM, assessed by bioelectrical impedance, and dual-energy x-ray absorptiometry (DXA) (Lunar DPX-IQ, USA) about one year after examination [30]. Pearson correlation coefficients between these two methods were 0.90 and 0.79 for the right and left arms, respectively; and 0.93 and 0.95 for the right and left legs, respectively.

### Assessment of education

The questions on education were included in the health interview. The information on education was combined into a variable describing three levels of education: low, middle and high. Persons who had no vocational training beyond a vocational course or on-the-job training, and who had not taken the matriculation examination, were classified as having a low level of education. Completion of vocational school was defined as a middle level of education. Furthermore, all those who had passed the matriculation examination, but who had no vocational training beyond a vocational course or on-the-job training, were also classified into this intermediate group. Subjects with high educational status comprised those with degrees from higher vocational institutions, polytechnics and universities.

### Statistical analysis

For the statistical analyses BMI, WHR and FFM were divided into sex-specific quintiles. High and low BMI, WHR and FFM were defined as extreme quintiles in the sex-specific distributions. The cut-offs for high BMI (30.1 kg/m^2 ^in men and 30.8 kg/m^2 ^in women) were consistent with the widely accepted cut-off BMI ≥ 30 kg/m^2 ^for obesity according to the World Health Organization [31]. There is no consensus with the WHR cut-offs. However, the cut-offs for high WHR (1.02 in men and 0.91 in women) were close to the proposed reference values or cut-off points used in previous studies: 1.00 for men and 0.90 for women [32] or 1.00 for men and 0.85 for women[33]. Furthermore, 0.99 for men and 0.88 for women were estimated to correspond a BMI of 30 in a Finnish study[19].

The associations of BMI, WHR and FFM with education were estimated using the general linear model[34]. The variations in standard deviations were studied using the distributions of the variables. Logistic regression analysis was used to examine the associations between education and the prevalence of low and high BMI, WHR and FFM[35]. In the first additive model, adjustment was carried out for age as a continuous variable and sex. In the second model, interaction terms between age and education and between sex and education were also included. Since significant interactions emerged, the main analyses were stratified by age and sex. Finally, BMI was also entered as a covariate in the additive models for FFM, and FFM was entered as a covariate in the additive models for BMI and WHR. All analyses were conducted using SAS 9.1.2 (SAS Institute, Inc., Cary, NC, USA).

## Results

Weight, height, body mass index (BMI), waist-to-hip ratio (WHR) and fat free mass (FFM) of the subjects are presented by sex and age in Table 1. In men, the mean weight, BMI and WHR increase with age until the age of 65 years, whereas the mean values of height and FFM decrease constantly with age. Similarly in women, the mean weight increases with age up to 65 years of age and the mean BMI up to 75 years of age. In women, however, the higher the age is, the lower are the mean values of height and FFM, and higher of WHR.

**Table 1:** Weight, height, body mass index, waist-to-hip ratio and fat free mass by age in men and women.

		Men			Women		
	Age	n	mean	**s.d**.	n	mean	**s.d**.
Weight, kg							
	30-44	1012	83.6	0.4	1097	68.3	0.4
	45-54	788	86.0	0.5	837	71.6	0.5
	55-64	509	85.5	0.6	590	72.6	0.5
	65-74	344	80.8	0.8	460	71.8	0.6
	75+	211	77.2	1.0	444	67.7	0.6
	30-64	2309	85.0	0.3	2524	70.2	0.4
	65+	555	79.6	0.7	904	70.5	0.3
	30+	2864	84.0	0.3	3428	70.4	0.2
Height, cm							
	30-44	1012	178.4	0.2	1097	165.0	0.2
	45-54	787	176.6	0.2	837	163.1	0.2
	55-64	209	175.0	0.3	589	161.1	0.2
	65-74	344	171.4	0.4	460	158.5	0.3
	75+	211	170.0	0.5	437	156.3	0.3
	30-64	2308	177.1	0.1	2523	163.4	0.2
	65+	555	171.0	0.3	897	157.6	0.1
	30+	2868	176.0	0.2	3420	162.0	0.1
Body mass index, kg/m^2^							
	30-44	1012	26.3	0.13	1097	25.1	0.15
	45-54	787	27.5	0.14	837	26.9	0.17
	55-64	509	27.9	0.18	589	28.0	0.19
	65-74	344	27.5	0.23	460	28.6	0.22
	75+	210	26.6	0.28	436	27.8	0.23
	30-64	2308	27.1	0.09	2523	26.4	0.10
	65+	554	27.2	0.19	896	28.3	0.16
	30+	2862	27.1	0.09	3419	26.9	0.08
Waist-to-hip ratio							
	30-44	1009	0.954	0.002	1097	0.836	0.002
	45-54	785	0.983	0.002	835	0.856	0.002
	55-64	505	0.987	0.003	587	0.867	0.003
	65-74	344	0.981	0.003	458	0.883	0.003
	75+	203	0.981	0.004	423	0.883	0.003
	30-64	2299	0.971	0.002	2519	0.883	0.002
	65+	547	0.981	0.002	881	0.850	0.001
	30+	2846	0.973	0.002	3400	0.858	0.001
Fat free mass, kg							
	30-44	974	66.8	0.3	1043	47.8	0.2
	45-54	74	66.4	0.3	796	47.9	0.2
	55-64	474	64.5	0.3	551	47.0	0.2
	65-74	307	59.6	0.5	411	45.3	0.3
	75+	166	56.0	0.7	316	42.8	0.3
	30-64	2202	66.2	0.2	2390	47.6	0.1
	65+	473	58.5	0.4	727	44.4	0.2
	30+	2675	64.9	0.2	3117	46.9	0.1

**Figure 1:**
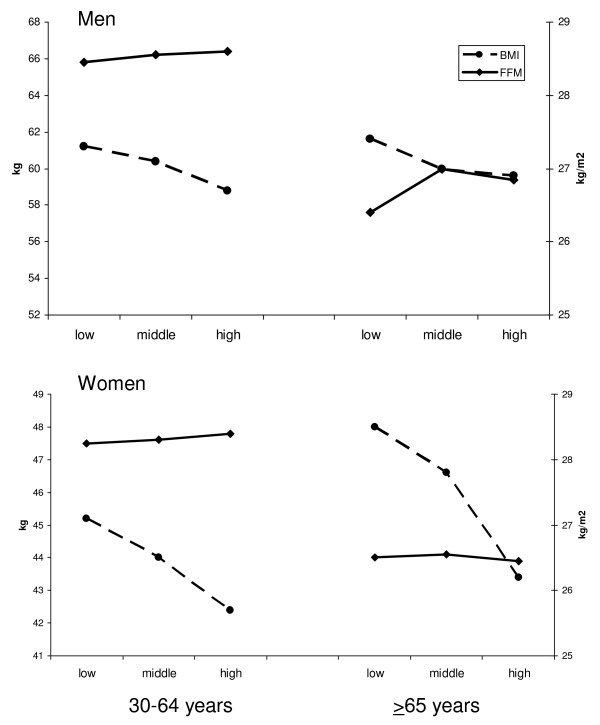
**The mean body mass index (kg/m^2^) and fat free mass (kg) in men (n = 2675) and women (n = 3117) by education**.

In the total study population, the mean BMI and WHR varied across educational groups both in men (Table 2) and women (Table 3). The values were highest among the least educated subjects, and lowest among the most educated subjects. In men, the educational gradient was observed in middle-aged (30-64 y.) men only (Table 2), whereas in women, the mean BMI and WHR varied by education in both age groups (Table 3). No differences in the mean FFM were observed in relation to education in the total study population (Tables 2 and 3). Nevertheless, the relation between the mean FFM and education seemed to vary by age and sex, such that the difference between educational groups in the mean FFM was statistically significant in older men (≥ 65 y) only. In this group, the mean FFM was lowest among the least educated men (Figure 1).

**Table 2:** The body composition by age and education in men.

		Level of education									
		Low			Middle			High			
Measurement	Age Group	n	mean	**s.d**.	n	mean	**s.d**.	n	mean	**s.d**.	p-value
**Body mass index, kg/m^2^**											
	30-64	703	27.3	4.4	972	27.1	4.0	633	26.7	4.1	0.03
	65+	394	27.2	4.1	106	27.0	3.9	54	26.9	2.9	0.90
	30+	1 097	27.3	4.3	1 078	27.1	4.0	687	26.7	4.0	0.02
**Waist-to-hip ratio**											
	30-64	702	0.98	0.06	967	0.97	0.06	630	0.96	0.06	<0.001
	65+	389	0.98	0.05	104	0.97	0.05	54	0.98	0.06	0.21
	30+	1 091	0.98	0.06	1 071	0.97	0.06	684	0.96	0.06	<0.001
**Fat-free mass, kg**											
	30-64	664	65.8	8.7	929	66.2	8.6	609	66.4	8.0	0.43
	65+	338	57.6	8.1	88	60.0	7.2	47	59.4	7.2	0.02
	30+	1 002	64.3	9.3	1 017	65.0	8.6	656	65.1	8.2	0.11

The mean values are adjusted for age (as a continuous variable) within each age group.

**Table 3:** The body composition by age and education in women.

		Level of education									
		Low			Middle			High			
Measurement	Age Group	n	Mean	**s.d**.	n	mean	**s.d**.	n	mean	**s.d**.	p-value
**Body mass index, kg/m^2^**											
	30-64	740	27.1	5.3	788	26.5	5.1	994	25.7	4.7	<0.001
	65+	660	28.5	4.9	145	27.8	4.9	88	26.2	4.0	<0.001
	30+	1 400	27.5	5.2	933	26.8	5.1	1 082	25.9	4.6	<0.001
**Waist-to-hip ratio**											
	30-64	739	0.86	0.06	787	0.85	0.06	992	0.84	0.06	<0.001
	65+	648	0.89	0.06	142	0.87	0.07	88	0.86	0.05	<0.001
	30+	1 387	0.87	0.06	929	0.86	0.06	1 080	0.84	0.06	<0.001
**Fat-free mass, kg**											
	30-64	699	47.5	5.9	749	47.6	6.0	941	47.8	6.0	0.67
	65+	530	44.0	5.7	118	44.1	5.4	77	43.9	4.8	0.95
	30+	1 229	46.8	6.0	867	46.7	6.0	1 018	46.8	6.0	0.96

The mean values are adjusted for age (as a continuous variable) within each age group.

The analysis of effect modification revealed that there were significant age-adjusted interactions between sex and education for low (the lowest quintile) BMI (*P *< 0.001) and low (the lowest quintile) FFM (*P *< 0.001). For the other outcomes the corresponding p-values varied between 0.15 and 0.47. In men, the interaction term between age and education was close to being statistically significant for high (the highest quintile) WHR (*P *= 0.08) and low FFM (*P *= 0.08), whereas in women, the effect modification by age was weaker. Due to these results, the final analyses for investigating associations of education with low and high body composition values were stratified into four groups: men and women aged 30-64 years or aged 65 years or older.

The age-adjusted associations between education and FFM were quite weak (Model 1, Table 4). No associations were observed in older age groups but in the middle-aged group, highly educated men were less likely to have a low FFM compared with men in the lowest education group. With adjustment for BMI, also men with middle level education and women with high level education in older groups were less likely to have a low FFM compared with their least educated counterparts (Model 2, Table 4).

**Table 4:** Odds ratios (OR) with 95% confidence intervals (95% CI) for low fat free mass (FFM) by education in middle-aged (30-64 years) and older (65 years and older) men and women

	Low FFM^1^				
Level of education		Model 1^2^		Model 2^3^	
	n	OR	95% CI	OR	95% CI
**Men 30-64 y**					
Low^4^(n = 664)	113	1.00		1.00	
Middle (n = 929)	123	0.78	0.59-1.04	0.73	0.53-1.00
High (n = 609)	70	0.67	0.48-0.93	0.52	0.36-0.75
**Men 65+ y**					
Low^4 ^(n = 338)	174	1.00		1.00	
Middle (n = 88)	35	0.65	0.40-1.07	0.57	0.33-0.99
High (n = 47)	20	0.69	0.37-1.29	0.61	0.30-1.23
**Women 30-64 y**					
Low^4 ^(n = 699)	106	1.00		1.00	
Middle (n = 749)	115	1.08	0.80-1.46	0.95	0.68-1.32
High (n = 941)	142	1.08	0.80-1.44	0.78	0.56-1.07
**Women 65+ y**					
Low^4 ^(n = 530)	195	1.00		1.00	
Middle (n = 118)	37	0.86	0.56-1.34	0.70	0.43-1.14
High (n = 77)	25	0.83	0.49-1.41	0.42	0.23-0.76

^1 ^The cut-offs for the lowest quintiles of FFM were in men <57.5 kg and in women <41.7 kg

^2^Model 1: Adjusted for age as a continuous variable in the additive model

^3 ^Model 2: Adjusted for age and BMI as continuous variables in the additive model

^4^The reference population

With adjustment for age, in middle-aged men, those with middle or high level education were less likely to have a high BMI compared with their less educated counterparts (Model 1, Table 5). Women with the highest level of education in both age groups were less likely to have a high BMI than women with low education (Model 1, Table 5). In middle-aged men, those with middle or high level education were less likely to have a high WHR than men with the lowest education (Model 1, Table 6). In both age groups, women with the highest level of education were less likely to have a high WHR than their least educated counterparts (Model 1, Table 6). In general, adjustment for FFM seemed to strengthen the associations of education with the prevalence of high BMI or high WHR (Model 2, Tables 5 and 6). For example, in the middle-aged group, men and women with middle level education seemed to be less likely to have a high BMI compared with their less educated counterparts. In women, this association reached statistical significance after adjustment for FFM.

**Table 5:** Odds ratios (OR) with 95% confidence intervals (95% CI) for high body mass index (BMI) by education in middle-aged (30-64 years) and older (65 years and older) men and women

	High BMI^1^				
Level of education		Model 1^2^		Model 2^3^	
	n	OR	95% CI	OR	95% CI
**Men 30-64 y**					
Low^4 ^(n = 703)	181	1.00		1.00	
Middle (n = 972)	181	0.77	0.60-0.99	0.69	0.52-0.92
High (n = 633)	92	0.57	0.43-0.76	0.45	0.32-0.63
**Men 65+ y**					
Low^4 ^(n = 394)	87	1.00		1.00	
Middle (n = 106)	19	0.74	0.43-1.29	0.48	0.24-0.95
High (n = 54)	11	0.88	0.43-1.79	0.75	0.33-1.69
**Women 30-64 y**					
Low^4 ^(n = 740)	188	1.00		1.00	
Middle (n = 788)	139	0.82	0.63-1.07	0.72	0.53-0.98
High (n = 994)	127	0.59	0.45-0.77	0.44	0.32-0.61
**Women 65+ y**					
Low^4 ^(n = 660)	185	1.00		1.00	
Middle (n = 145)	35	0.80	0.53-1.22	0.66	0.39-1.10
High (n = 88)	9	0.29	0.14-0.59	0.22	0.10-0.52

^1^The cut-offs for the highest quintiles of BMI were in men ≥ 30.05 kg/m^2 ^and in women ≥ 30.77 kg/m^2^

^2^Model 1: Adjusted for age as a continuous variable in the additive model

^3 ^Model 2: Adjusted for age and FFM as continuous variables in the additive model

^4^The reference population

**Table 6:** Odds ratios (OR) with 95% confidence intervals (95% CI) for low and high waist-to-hip ratio (WHR) by education in middle-aged (30-64 years) and older (65 years and older) men and women

	High WHR^1^				
Level of education	n	Model 1^2^		Model 2^3^	
		OR*	95% CI*	OR^≠^	95% CI^≠^
**Men 30-64 y**					
Low^4 ^(n = 702)	200	1.00		1.00	
Middle (n = 967)	179	0.71	0.56-0.90	0.66	0.51-0.85
High (n = 630)	74	0.41	0.30-0.56	0.35	0.26-0.49
**Men 65+ y**					
Low^4 ^(n = 389)	90	1.00		1.00	
Middle (n = 104)	15	0.56	0.31-1.01	0.45	0.23-0.88
High (n = 54)	10	0.75	0.36-1.56	0.71	0.33-1.54
**Women 30-64 y**					
Low^4 ^(n = 739)	175	1.00		1.00	
Middle (n = 787)	129	0.81	0.62-1.06	0.75	0.57-1.01
High (n = 992)	89	0.43	0.32-0.57	0.38	0.27-0.51
**Women 65+ y**					
Low^4 ^(n = 648)	231	1.00		1.00	
Middle (n = 142)	40	0.71	0.48-1.06	0.73	0.47-1.15
High (n = 88)	14	0.34	0.19-0.62	0.32	0.16-0.62

^1^The cut-offs for the highest quintiles of WHR were in men ≥ 1.02 and in women ≥ 0.91

^2^Model 1: Adjusted for age as a continuous variable in the additive model

^3 ^Model 2: Adjusted for age and FFM as continuous variables in the additive model

^4^The reference group

## Discussion

The association between education and FFM was fairly weak. Highly educated men, however, were less likely to have a low FFM suggesting that highly educated individuals have more muscles compared with their less educated peers. This association was not explained by differences in BMI as after adjustment for BMI, the associations remained in the middle-aged men and strengthened in older men and women. Not surprisingly, educational level was inversely associated with overall (high BMI) and abdominal (high WHR) obesity both in men and women. FFM was not found to explain these educational gradients but adjustment for FFM seemed to slightly strengthen the inverse associations between education and obesity.

To our knowledge, BIA based data on body composition in representative population sample have not earlier been reported. More importantly, to date, the present study is the first one that has investigated whether there is any relation between FFM and educational level.

Our findings that education was inversely associated with the prevalence of overall obesity both in men and women are consistent with many earlier studies both in Finland [11,12,14] and other countries[7-10,13]. In agreement with our results, the findings have been more consistent in women than in men[10,12,14,15]. In addition, in most of the previous studies, central obesity has also been shown to be inversely associated with education both in men and women [14,16-21].

In the majority of the earlier studies concerning the association between education and obesity, the subjects have been mainly under 65 years of age. In the present study, the inverse associations between education and both overall and abdominal obesity were evident in older women but not in older men, which supports the findings of a recent study from Spain, [36] where inverse associations between education and BMI and waist circumference were evident in older (≥ 60 year) women but not in older men. Contrary to our findings, another study from Chicago [37] found BMI to be inversely associated with education both in men and women in older (≥ 64 year) groups.

The reasons for gender differences in the educational patterning of obesity by age are not known. The association between socio-economic status and obesity is complex: it is bidirectional and confounded by other factors such as heredity, [38] health behaviour [25,39] or in general social and cultural norms[15]. It is possible that a strong desire to be thin among the highest educated women [25,39] lasts throughout their entire lives. It is also possible that a few decades ago, obesity and especially abdominal obesity was a sign of high socio-economic status in men and was thus a more acceptable characteristic for men. This may be the reason for the diminished association between obesity and education among the older men.

In older men, the standard deviation in FFM varied across educational groups, being largest in the least educated men, and narrowest in the highly educated men. This finding indicates larger heterogeneity in body composition in the least educated older men. It is possible that due to this heterogeneity, the association between FFM and education has diminished. It is also worth of noticing that despite the lack of educational gradient in BMI for older men, the mean FFM was smaller in men with low education compared with their more educated counterparts. This finding suggests that those men may have more adverse body composition. One explanation for this might be more frequent co-morbid conditions (e.g. heart failure or chronic obstructive pulmonary disease) that have been shown to be related to lower FFM[40,41]. Contrary to this assumption, however, the previous Finnish study showed lower socio-economic status to be associated with smaller waist circumference in working-aged men when BMI was adjusted for[17]. In all, socio-economic patterning of obesity has shown to be divergent. For example, being thin, as defined by low BMI, has been observed to be associated with unemployment and low income in men [42,43] and with low income in women[42].

Contrary to our expectations, there was no evidence that high FFM is a characteristic of people with low educational status. Our findings did not support our hypothesis of less educated people due to manual work to have higher FFM than people with higher education. Our results do indeed suggest that less-educated middle-aged individuals have less muscle compared with their more educated peers. Compared with the working conditions of the early 1980s, the typical jobs today are physically much less demanding[26]. Physical activity at work decreased, whereas leisure-time activity increased in Finland between the 1980s and the 1990s[19,26]. In all, during the same period, total physical activity decreased[26].

We assumed that people carrying out more physical activity have higher FFM than people carrying out less physical activity. However, the association between physical activity and FFM is not so simple. The type of exercise, aerobic or resistance exercise, may have different impacts on FFM[44]. Furthermore, physical activity has been observed to be positively [16] or inversely [45] associated with educational level in men. In Eastern Finland in the 1970s, people carrying out a lot of physical activity were better educated than people who did not carry out much physical activity.46] In the early 1980s, this educational gradient disappeared in both genders, but in 1997 men who did not carry out much physical activity were better educated than those who did. It has also been observed that high socio-economic status or high level of education is associated with less job-related physical activity [25,47] but more leisure time fitness activity[25,27,47]. It may be that several decades ago, less educated, manual workers probably did have more muscles compared with higher educated people, whereas nowadays highly educated people who may be more physically active in their leisure time may be more muscular.

The variation in FFM could not explain the association of education with overall and abdominal obesity but it strengthened these educational gradients to some extent. This was somewhat unexpected, because FFM is one of two important body composition components (i.e., fat mass and FFM), which influence both BMI and WHR although it has less influence on WHR. Men generally have a higher mean FFM than women, who in turn have higher fat mass and fat percentage than men [48-50]. Furthermore, BMI cannot distinguish between fat mass and FFM, and increased WHR may reflect decreased muscle mass in the lower part of the body as much as increased abdominal adiposity[51,52]. Therefore we assumed that FFM could confound and explain the different association between education and obesity among men and women. Indeed, our results of FFM strengthening the associations may suggest that differences in overall obesity defined by BMI and abdominal obesity defined by WHR indicate quite well differences in fat mass.

The strengths of our study include a database with a large number of subjects and trained personnel. A further strength was that a comprehensive set of methods was used to measure body composition.

Regardless of the large number of subjects in our study, there were only a few subjects in the older age groups, especially men, in the middle and high educational level after dividing subjects into the lowest and highest quintiles of BMI, WHR and FFM. For these kinds of analyses sample size should be even larger to give the analyses more statistical power.

The field conditions of the health examination were standardized as far as possible[28,29]. All the subjected had been asked to come to the examination after fasting on the same day. However, the examinations were started between 8 o'clock in the morning and 2 o'clock in the afternoon, sometimes even later, so that BIA results which depend on the time of day and the duration of fasting may vary because of variations in the conditions.

In the future, possible confounding factors such as physical activity, eating habits and chronic diseases should be taken into account to improve understanding of association between deviant body composition and education. In addition, prospective studies should give more information on causes and consequences of deviant body composition and education.

## Conclusions

The inverse association of educational level with overall and abdominal obesity cannot be explained by FFM. Adjustment for FFM may even strengthen the educational gradient. Contrary to our first hypothesis, high FFM is not a characteristic of people with a low level of education.

## Competing interests

The authors declare that they have no competing interests.

## Authors' contributions

ESN participated in the design of the study and conducting data analyses, and wrote the first draft of the paper. MLK, SM and MH designed the study and participated with interpretation of the data and writing of the paper. PK participated in the study design and interpretation of the data, and helped with the statistical analyses. HR performed the statistical analyses. AA is the director of the Health 2000 Study and acquired the data. All authors read and approved the final manuscript.

## Pre-publication history

The pre-publication history for this paper can be accessed here:

http://www.biomedcentral.com/1471-2458/9/448/prepub
